# First report of *Meloidogyne hapla* on hemp (*Cannabis sativa*) in Oregon

**DOI:** 10.2478/jofnem-2024-0008

**Published:** 2024-03-14

**Authors:** Lester Núñez-Rodríguez, Hannah Rivedal, Amy Peetz, Cynthia M. Ocamb, Inga Zasada

**Affiliations:** Department of Botany and Plant Pathology, Oregon State University, Corvallis, OR 97331; USDA-ARS Forage Seed and Cereal Research Unit, Corvallis, OR 97331; USDA-ARS Horticultural Crops Disease and Pest Management Research Unit, Corvallis, OR 97331

**Keywords:** *Cannabis sativa*, detection, hemp, identification, *Meloidogyne hapla*, northern root-knot nematode, PCR-RFLP, sequencing, specific-primers

## Abstract

Hemp is a crop that has gained interest in Washington and Oregon. As with other crops, hemp production faces challenges due to biotic factors, including plant-parasitic nematodes. During a survey for plant-parasitic nematodes associated with hemp, *Meloidogyne* sp. was found in a composite root sample collected in Oregon. Morphological characterization of second-stage juveniles identified the nematode as *Meloidogyne hapla*. Molecular identification confirmed the population as *M. hapla*. To our knowledge, this is the first report of *M. hapla* on hemp in the Pacific Northwest of the United States.

The Pacific Northwest (PNW) of the United States (Idaho, Oregon, and Washington) encompasses a wide range of ecoclimates, which allows for the production of a wide diversity of crops (USDA-NASS, 2019). A crop that has recently gained importance in Washington and Oregon is hemp (*Cannabis sativa*). In 2021, total hemp production in these states generated more than $249 million (USDA-NASS, 2022). However, as with many other crops, hemp production faces challenges due to pests and diseases, including plant-parasitic nematodes. Endoparasitic nematodes top the list of the most important plant-parasitic nematodes and include *Meloidogyne*, arguably the most important plant-parasitic nematode globally (Jones et al., 2013). This nematode can parasitize more than 2,000 plant species, including non-agricultural and economically important agricultural crops (Agrios, 2005; Núñez-Rodríguez et al., 2022). Despite the growing importance of hemp in the PNW, there is little information about plant-parasitic nematodes associated with the crop in this region (Núñez-Rodríguez et al., 2023).

In 2022, during a survey of plant-parasitic nematodes in the PNW, *Meloidogyne* sp. was found in a composite root sample of hemp ‘Suver Haze’ (21,336 second-stage juveniles/100 g of wet root tissue) from a field located in Benton County, Oregon. No galls were observed on the hemp root sample. *Meloidogyne* sp. was extracted from roots under intermittent mist (Zasada et al., 2015). Second-stage juveniles (J2) (n = 15) were hand-picked for morphological characterization. Nematodes were temporarily mounted, photographed, and measured using an Olympus BX51 microscope (Melville, NY) with an Olympus DP72 camera (Center Valley, PA). The following measurements (mean μm ± standard deviation) were determined: body length was 429.6 ± 16.5 (Fig. 1A), body width 14.6 ± 0.4, stylet was weak with a length of 13.9 ± 0.3 and knobs were rounded (Fig. 1B), anal body diameter was 10.7 ± 0.4, tail with length was 61 ± 2.5 with different shapes (Figs. 1C,D), and hyaline region length was 14.2 ± 1.4. Measurements of the *Meloidogyne* sp. population collected from Oregon hemp roots were within the reported range for *Meloidogyne hapla* (Hunt and Handoo, 2009; Meressa et al., 2015; Sohrabi et al., 2015; Akyazi et al., 2017).

**Figure 1:**
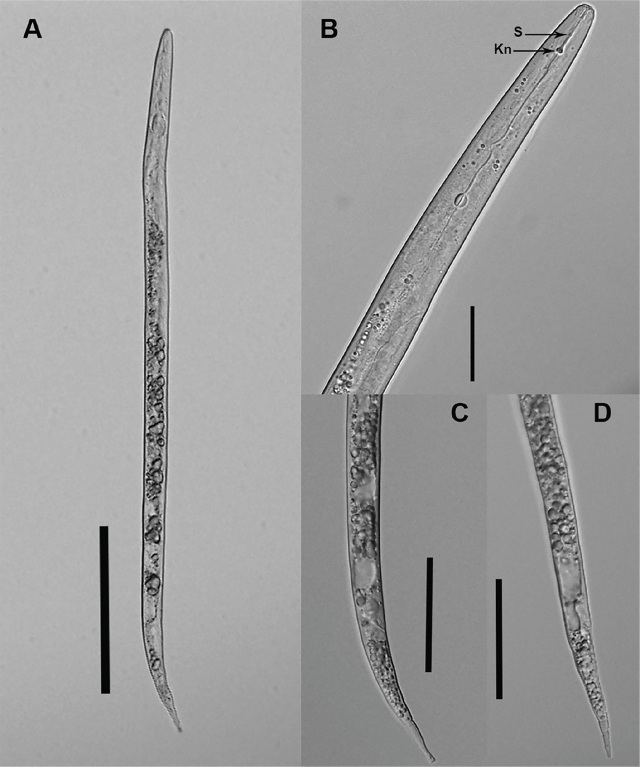
*Meloidogyne hapla*, A) body length, B) stylet (S) and knobs (Kn), C) and D) tail. Scales: A: 100 μm, B: 20 μm, and C,D: 50 μm.

To confirm the identification of *M. hapla*, DNA was extracted from single J2 (n = 8). Each nematode was hand-picked and cut for DNA extraction (Peetz and Zasada, 2016). Primers C2F3 and 1108 were used to amplify the mitochondrial DNA COXII (Powers and Harris, 1993). Amplification of the mitochondrial DNA resulted in a single band of ~500 bp. PCR products were cleaned up using the enzyme ExoSap-IT (Thermo Fisher Scientific, USA) and sent for bidirectional sequencing with the same primers used for amplifications at the Oregon State University Center for Quantitative Life Science (Corvallis, OR). The analysis of the sequences resulted in three haplotypes (GenBank accession numbers OQ920919–OQ920921). The BLASTn analysis of these sequences matched with *M. hapla* with a percentage of identity from 95.1% (accession KP681262) to 100% (accession AY757899). The phylogenetic relationship of *M. hapla* and other *Meloidogyne* spp. sequences retrieved from GenBank was estimated using the Bayesian analysis method (Larget and Simon, 1999). The resulting phylogenetic tree placed the *M. hapla* sequences from hemp in Oregon with other sequences of *M. hapla* with a posterior probability value of 100% (Fig. 2), supporting the results described above.

**Figure 2:**
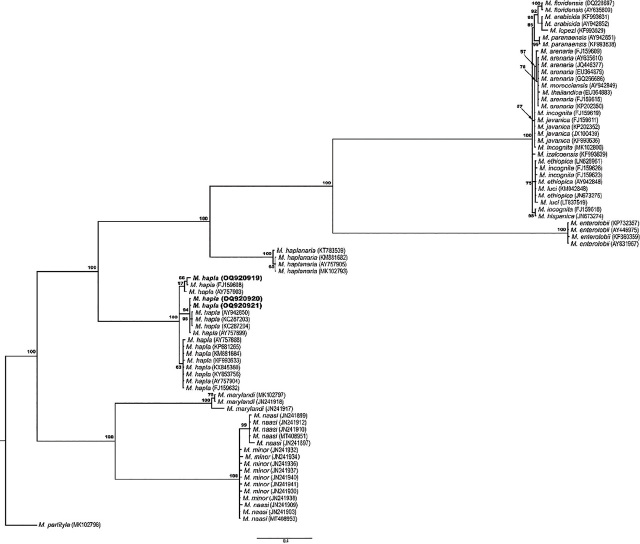
Phylogenetic relationships between *Meloidogyne* species as inferred from the Bayesian analysis of the mitochondrial DNA *CoxII*-IGS under the GTR + I + G model. Posterior probabilities of over 50% are given for appropriate clades. Sequences generated in this study are identified in bold. *Meloidoyne hapla* sequences were retrieved from GenBank, aligned, and trimmed with BioEdit v.7.0.5.3. The best substitution model was determined using jModelTest 2.1.10 v20160303 and the Bayesian analysis was performed using MrBayes v3.2.6. Finally, the tree was visualized using FigTree v1.4.3.

These results were also confirmed by using PCR-RFLP and PCR-species specific Intergenic Spacer primers. For PCR-RFLP, the enzyme *DRAI* (Thermo Fisher Scientific) was used to digest the PCR product obtained with the primers C2F3 and 1108. The restriction pattern consisted of two bands (approximately 200 bp and 250 bp) corresponding to the pattern described for *M. hapla* (Powers and Harris, 1993). The species was confirmed with a multiplex PCR using the species-specific primers JMV1 (5′-GGATGGCGTGCTTTCAAC-3′), JMV2 (5′-TTTCCCCTTATGATGTTTACCC-3′) and JMVhapla (5′-AAAAATCCCCTCGAAAAATCCACC-3′), which yielded a band of 440 bp (data not shown) corresponding to *M. hapla* (Wishart et al., 2002).

The pathogenicity of *M. hapla* on hemp was evaluated under greenhouse conditions. Because of the proprietary nature of many hemp varieties, ‘Sauver haze’ was not available for evaluation in greenhouse studies; therefore, hemp ‘Alpha Explorer’ was considered. Seedlings (5-week-old) of this variety were planted in pots containing 2.1 kg of steam-pasteurized 1:1 sand:loam soil mix. Five hemp plants were inoculated with 4,000 eggs, and five hemp plants were non-inoculated and included as negative controls; three 4-week-old tomato ‘Rutgers’ plants were inoculated with 1,100 eggs as positive controls. The experiment was conducted twice. The experiment was conducted in a greenhouse with average temperatures of 20.8 ± 3.2 °C, and plants were harvested 60 days after inoculation. Eggs from roots and J2 from soil were extracted with the NaOCl and Baermann funnel methods, respectively (Hussey and Barker, 1973; Zasada et al., 2015). Plants did not show any differences in growth based on plant biomass between treatments. No galls were observed on the roots of the inoculated hemp plants. The reproduction factor (RF) was calculated by dividing the final population density by the initial population density. Results from both experiments were combined since there was no statistical difference between the trials (*P* > 0.05). *Meloidogyne hapla* successfully reproduced on the tomato positive control (RF = 208.5 ± 18.3), while hemp was a poor host (RF = 0.6 ± 0.1). This result contradicted the high population densities of *M. hapla* observed in the roots of hemp plants collected from the field. This may be explained by differences in host suitability of hemp genotypes. For example, de Meijer (1993) tested 123 hemp cultivars and reported varying resistance levels to *M. hapla*, which ranged from highly resistant to moderately susceptible.

This is the first report of *M. hapla* on hemp in the PNW. *Meloidogyne hapla* was previously found on hemp in Iowa in 1966 (Norton, 1966). Studies should be conducted to determine pathogenicity of *M. hapla* on a range of hemp cultivars and the role of hemp as a rotation crop for the management of *M. hapla* in the PNW.
